# Topical β-Caryophyllene for Dermatologic Disorders: Mechanisms, Human Evidence, and Clinical Translation

**DOI:** 10.3390/ph18111605

**Published:** 2025-10-23

**Authors:** Amina M. Bagher

**Affiliations:** Department of Pharmacology and Toxicology, Faculty of Pharmacy, King Abdulaziz University, Jeddah 21589, Saudi Arabia; abagher@kau.edu.sa; Tel.: +966-547187973

**Keywords:** β-caryophyllene, CB_2_ receptor, endocannabinoid system, topical cannabinoids, skin inflammation, oxidative stress, wound healing, dermatology

## Abstract

Background: Chronic inflammatory skin disorders, including atopic dermatitis, psoriasis, acne, and chronic wounds, affect nearly two billion people worldwide, impose substantial morbidity and economic burden, and remain only partially controlled by existing therapies. The cutaneous endocannabinoid system (ECS), comprising cannabinoid receptors, endocannabinoids, and their metabolic enzymes, regulates inflammation, pruritus, barrier integrity, and tissue repair; cannabinoid receptor type 2 (CB_2_) has emerged as a particularly relevant target. β-Caryophyllene (BCP), a dietary sesquiterpene and highly selective CB_2_ agonist with favorable safety and pharmacokinetic attributes, has attracted attention as a promising topical candidate. Methods: We systematically searched PubMed, Embase, and Web of Science (inception–30 July 2025) for studies on “β-caryophyllene” and dermatological outcomes, prioritizing purified BCP and analytically characterized BCP-rich fractions. Quantitative parameters, including tested concentration ranges (0.5 µM–10%) and principal mechanistic outcomes, were extracted to provide a translational context. Results: BCP penetrates the stratum corneum, suppresses NF-κB/MAPK and IL-4/TSLP pathways, enhances Nrf2-driven antioxidant defenses, and accelerates re-epithelialization and collagen remodeling. Across in vitro, in vivo, and formulation studies, BCP produced consistent anti-inflammatory and barrier-restorative effects within this concentration range. CB_2_ antagonism attenuated these responses, confirming receptor specificity. BCP’s volatility and autoxidation to β-caryophyllene oxide (BCPO) necessitate stability-by-design strategies using antioxidants, low-oxygen processing, and protective packaging. Human evidence, limited to BCP-rich botanicals such as Copaifera oleoresins, suggests benefits for scars, wounds, and acne but lacks compound-specific validation. Conclusions: BCP exhibits coherent CB_2_-mediated anti-inflammatory, antipruritic, antioxidant, and reparative actions with a favorable safety profile. Dose-defined, oxidation-controlled clinical trials of purified BCP are warranted to establish its potential as a steroid-sparing topical therapy.

## 1. Introduction

Chronic and inflammatory skin disorders are among the most common non-fatal conditions worldwide and impose a substantial clinical and economic burden. In the Global Burden of Disease 2019 analysis, skin and subcutaneous diseases ranked fourth in years lived with disability, affecting an estimated 1.79 billion people [1]. High-burden conditions include atopic dermatitis, psoriasis, acne, urticaria, and chronic wounds [2]. In Middle Eastern settings, particularly Saudi Arabia, eczema/dermatitis and acne are the most common conditions leading to outpatient dermatology visits and are associated with significant psychosocial consequences [3,4,5].

Standard topical agents, including corticosteroids, calcineurin inhibitors, vitamin D analogs, and antimicrobials, remain the cornerstone of therapy. However, chronic use is constrained by local adverse effects, antimicrobial resistance, and adherence challenges, while psoriasis often requires escalation to costly biologics [6,7,8]. More recently, non-steroidal agents, such as phosphodiesterase-4 and Janus kinase inhibitors, have broadened treatment options; however, their high cost, restricted access, and the need for long-term safety monitoring limit their widespread use [9,10]. These limitations have intensified interest in phytochemicals that target shared inflammatory and barrier pathways, with the potential to complement or partially substitute conventional therapies.

The cutaneous endocannabinoid system (ECS), comprising cannabinoid receptors CB_1_ and CB_2_, the endocannabinoids anandamide (AEA) and 2-arachidonoylglycerol (2-AG), and metabolic enzymes such as fatty acid amide hydrolase (FAAH) and monoacylglycerol lipase (MAGL), is increasingly recognized as a key regulator of skin physiology. It orchestrates keratinocyte proliferation and differentiation, immune tone, nociception, and barrier homeostasis [11,12]. Dysregulation of this system is evident across primary dermatoses, including reduced AEA/2-AG and CB_1_/CB_2_ expression in atopic dermatitis, pro-inflammatory receptor–enzyme shifts in psoriasis, CB_2_–mitogen-activated protein kinase (MAPK)-driven lipogenesis in acne, and impaired re-epithelialization when CB_2_ tone is diminished during wound repair. Collectively, these alterations nominate CB_2_ as a peripheral control node for inflammation, pruritus, and repair [13,14].

Reflecting this mechanistic rationale, cannabinoid-based preparations, most notably cannabidiol (CBD) and palmitoylethanolamide (PEA), have been evaluated in dermatologic conditions such as atopic dermatitis, psoriasis, pruritus, and acne, with early studies suggesting symptomatic improvement [15,16,17,18]. However, both CBD and PEA act through multiple, partly overlapping receptor systems, including CB_1_ and peroxisome proliferator–activated receptor alpha (PPARα), leading to variable efficacy and unpredictable off-target effects [12,19,20]. In contrast, β-caryophyllene (BCP) is a highly selective CB_2_ agonist that directly engages the peripheral anti-inflammatory axis of the endocannabinoid system, offering a mechanistically defined, non-psychotropic, and targeted therapeutic approach [21,22,23]. This selectivity addresses a significant unmet need for safe, non-steroidal agents that can restore cutaneous immune balance and barrier integrity [6,7,8,9].

Significantly, CB_2_ receptors are enriched on peripheral immune cells, keratinocytes, fibroblasts, sebocytes, and small-diameter sensory fibers, while their expression in the central nervous system (CNS) remains minimal [24]. This peripheral bias enables selective CB_2_ agonists to exert anti-inflammatory and antipruritic effects without the psychotropic risks associated with CB_1_ activation [25,26]. Topical delivery further enhances this selectivity by confining exposure to the skin and minimizing systemic uptake, supporting a steroid-sparing strategy applicable to atopic dermatitis, psoriasiform inflammation, sebaceous disorders, and impaired wound healing.

Within this therapeutic landscape, BCP emerges as a promising candidate. BCP is a dietary sesquiterpene and highly selective CB_2_ agonist with negligible CB1 activity, abundant in essential oils of black pepper, clove, oregano, copaiba, and Cannabis sativa [21,27]. It is recognized as Generally Recognized as Safe (GRAS) for ingestion (FEMA No. 2252) and listed by the U.S. Food and Drug Administration (FDA) for food use; however, these designations do not establish topical efficacy or dermal safety [28,29]. Its physicochemical profile, characterized by a molecular weight below 500 Da and high lipophilicity, favors partitioning into the stratum corneum and adnexal pathways, aligning with the pharmacological requirements of topical therapy [12].

Preclinical studies support these mechanistic expectations. In atopic and irritant dermatitis models, topical BCP suppresses keratinocyte interleukin-4 (IL-4)–driven thymic stromal lymphopoietin (TSLP) signaling and improves lesion severity [30]. In wound and post-procedural models, BCP accelerates re-epithelialization and collagen remodeling, effects attenuated by CB_2_ antagonism, consistent with target engagement [31,32,33,34]. Early human-relevant signals, such as relief of sunburn-related pain, have been observed with BCP-rich copaiba mixtures, though attribution to BCP itself remains provisional [35]. However, BCP is not without caveats: high-dose epicutaneous exposure can provoke itch in mice [36], and CB_2_-mediated lipogenesis raises concerns for acne-prone skin [37]. These complexities underscore the need for dose optimization and rational combinations.

Translation progress is further constrained by chemistry. BCP is volatile and prone to autoxidation, yielding products such as caryophyllene oxide (BCPO) that alter pharmacodynamic (PD) properties and may increase the risk of sensitization [38,39,40]. Stability-by-design approaches, including the use of antioxidants, low-oxygen processing, and oxygen- and UV-barrier packaging, are therefore essential. Because on-skin exposure is dictated as much by formulation as by dose, development must integrate in vitro release testing (IVRT), in vitro permeation testing (IVPT), and depth-resolved tape stripping to link applied concentration, dermal levels, and pharmacological response while confirming minimal systemic uptake.

This review uniquely positions BCP as a next-generation, CB_2_-selective phytocannabinoid for dermatologic use, distinguished from existing multi-target cannabinoids by its mechanistic precision, peripheral safety profile, and formulation-dependent translatability. It synthesizes mechanistic, preclinical, and early clinical evidence, distinguishes purified BCP from BCP-rich mixtures, and critically examines formulation, safety, and regulatory considerations to identify credible translational routes for advancing BCP from a phytochemical of interest to a drug-quality dermatologic therapy.

## 2. Cannabinoid Receptors and the Endocannabinoid System in the Skin

### 2.1. Overview of the Cutaneous Endocannabinoid System

In the skin, the ECS functions as a locally gated, stimulus-responsive lipid signaling network, with CB_1_ and CB_2_ receptors mapped to basal and suprabasal keratinocytes, Langerhans/dendritic cells, mast cells, fibroblasts, melanocytes, sebocytes, eccrine glands, hair-follicle epithelia, dermal vasculature, and small-diameter sensory fibers [12,13]. eCBs are generated “on demand” and cleared near their site of action, creating steep pericellular gradients that gate short-range autocrine and paracrine communication. CB_1_ and CB_2_ are class A G-protein-coupled receptors (GPCRs) that primarily couple to Gi/o proteins; upon activation, they inhibit adenylyl cyclase, regulate MAPK cascades, and modulate multiple ion channels [11]. Signaling through Gβγ also engages the phosphoinositide 3-kinase (PI3K)/protein kinase B (AKT) signaling pathway. Moreover, tunes Ca^2+^/K^+^ channel activity, providing fine control over epidermal programs and mediator release [12,13].

AEA is produced predominantly from N-acyl-phosphatidylethanolamine (NAPE) via NAPE-specific phospholipase D (NAPE-PLD), which was cloned and functionally characterized in 2003–2004 [41]. 2-AG is generated “on demand” from diacylglycerol by sn-1–specific diacylglycerol lipases DAGLα and DAGLβ [42]. Degradation is predominantly catalyzed by FAAH for AEA and by MAGL for 2-AG [43,44]. Additional hydrolases, such as α/β-hydrolase domain–containing proteins 6 and 12 (ABHD6 and ABHD12), contribute to the spatially restricted turnover of 2-AG [45]. Ligand availability is further shaped by intracellular carriers, notably fatty-acid binding proteins (FABP5/7), which chaperone AEA to FAAH for degradation [46].

In cutaneous tissues, eCBs are produced in response to mechanical stress, thermal change, microbial products, cytokines, or neuropeptides and are rapidly degraded to maintain baseline tone. Together, this keeps signaling tightly local: eCBs act over very short distances as autocrine (to the same cell) and paracrine (to neighboring cells) cues at the epidermal–immune–neuronal interface, rather than driving tissue-wide effects. Because the enzymes that synthesize (NAPE-PLD, DAGLα/β) and degrade (FAAH, MAGL, ABHD6/12) eCBs are localized in discrete cellular zones, the signal remains confined to small membrane/tissue microdomains and does not spread laterally [12,13].

Beyond CB_1_ and CB_2_, eCBs and related lipids modulate a range of non-cannabinoid targets, including transient receptor potential (TRP) channels, peroxisome proliferator–activated receptors (PPARs), and orphan GPCRs such as G protein-coupled receptor 55 (GPR55), transient receptor potential vanilloid 3 (TRPV3), 4 (TRPV4), and transient receptor potential melastatin 8 (TRPM8), are notable TRP family members influenced by AEA and other lipid mediators; AEA can activate TRPV1 and inhibit TRPM8, while related N-acylethanolamines and monoacylglycerols modulate TRPV3 and TRPV4 [47,48,49]. These Ca^2+^-permeable channels integrate thermal, osmotic, mechanical, and chemical stimuli, translating them into changes in epidermal calcium homeostasis and downstream programs of barrier formation, inflammation, pain, and itch [50,51,52,53]. As such, TRPs and other non-cannabinoid targets are now recognized as integral parts of the “expanded ECS” or endocannabinoidome, broadening the pharmacological landscape for ECS modulation in skin biology [47,54].

### 2.2. Cellular Expression and Functions of the Endocannabinoid System in the Skin

**Figure 1:**
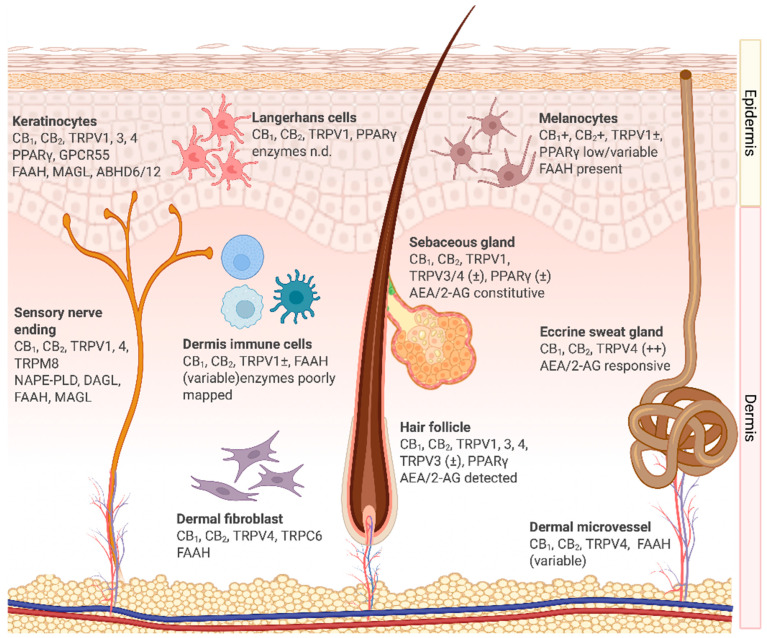
Distribution of endocannabinoid system components in human skin. Abbreviations: 2-AG, 2-arachidonoylglycerol; ABHD6/12, α/β-hydrolase domain–containing proteins 6 and 12; AEA, N-arachidonoyl ethanolamide (anandamide); ABHD6/12, α/β-hydrolase domain–containing proteins 6 and 12 CB_1_, cannabinoid receptor type 1; CB_2_, cannabinoid receptor type 2; DAGL, diacylglycerol lipase; FAAH, fatty acid amide hydrolase; GPR55, G-protein–coupled receptor 55; MAGL, monoacylglycerol lipase; n.d., not determined; NAPE-PLD, N-acyl phosphatidylethanolamine phospholipase D; PPARγ, peroxisome proliferator-activated receptor gamma; TRPC6, transient receptor potential canonical channel 6; TRPM8, transient receptor potential melastatin 8; TRPV1–4, transient receptor potential vanilloid channels 1 to 4. Figure modified from [56] and Created in BioRender. Bagher, A. (2025) https://BioRender.com/h3e28sb.

Extensive histological, biochemical, and functional evidence confirms that the ECS spans the epidermis, adnexal structures, dermal vasculature, pigmentary units, and resident neuro-immune elements, integrating sensory, immune, barrier, and regenerative control. The localization of ECS components is summarized in Figure 1, which illustrates the expression of receptors, ligands, and enzymes across major human skin compartments. This distributed expression highlights the capacity of eCB signaling to coordinate keratinocyte differentiation, immune cell activation, and neurocutaneous communication in a compartment-specific manner. Importantly, altered expression of CB_1_, CB_2_, TRP channels, and metabolic enzymes has been observed in inflammatory dermatoses, underscoring their functional relevance to disease pathophysiology [15,55].

Keratinocytes, the primary cells of the epidermis, express CB_1_ and CB_2_, along with the complete enzymatic machinery for AEA and 2-AG turnover, including FAAH, MAGL, and ABHD6/12, which enables local synthesis, uptake, and degradation. CB1 activation downregulates stress-response keratins K6 and K16, thereby attenuating hyperproliferative programs [57]. CB/FAAH/MAGL pathways influence differentiation, while TRPV4 promotes the assembly of adherens and tight junctions, thereby accelerating barrier recovery after insults [51,53]. Robust TRPV1 activation can transiently delay repair, integrating noxious thermal or chemical inputs into the maintenance of barrier homeostasis [12]. GPR55 is detectable in keratinocyte lineages and is up-regulated in squamous-cell carcinoma, where it engages extracellular signal–regulated kinase (ERK), AKT, and Ras homolog family member A (RhoA) pathways to drive proliferation [58]. Collectively, keratinocytes act as both producers and responders within the eCB network, coupling barrier integrity to immune surveillance.

Langerhans cells, the resident antigen-presenting cells of the epidermis, express the CB_2_ receptor. Activation reduces the release of interleukin 12 (IL-12) and tumor necrosis factor-α (TNF-α), downregulates major histocompatibility complex (MHC) class II and co-stimulatory molecules (CD80/CD86), and suppresses T-cell priming 52 [12,59,60]. In parallel, activation of TRPV1 by AEA inhibits maturation and further reduces IL-12 production, fostering a tolerogenic state. Through these mechanisms, CB_2_ and TRPV1 regulate the balance between tolerance and activation, which is clinically relevant to atopic dermatitis and allergic contact dermatitis.

Immune cells of the dermis, including dendritic cells, macrophages, and mast cells, represent key targets of cannabinoid signaling in the skin. Dendritic cells in the dermis use CB_2_ activation to suppress pro-inflammatory cytokines such as TNF-α, IL-12, and interleukin 23 (IL-23), thereby skewing the immune milieu toward resolution [12,26,61]. TRPV1, conversely, contributes to maturation and antigen presentation [47,48].

Macrophages express CB_1_ and CB_2_. CB_2_ engagement reduces TNF-α and interleukin 1β (IL-1β), limits M1-like polarization, and promotes resolution, in part through the upregulation of IL-10 [14,62]. These effects are relevant to wound healing and chronic inflammatory states. Mast cells, enriched around hair follicles, express CB_1_ and CB_2_. CB_1_ signaling inhibits maturation and degranulation, reducing perifollicular inflammation [63,64]. Collectively, these immune compartments demonstrate that CB_2_/CB/TRP cross-talk is a central regulator of cutaneous inflammatory tone.

Sensory nerve endings within the epidermis and dermis express CB_1_, CB_2_, and several TRP channels, particularly TRPV1 and TRPV4. CB_1_ activation modulates nociceptive transmission and reduces neuropeptide release, including substance P and calcitonin gene-related peptide, thereby attenuating neurogenic inflammation and pruritus [65,66]. TRPV1 mediates noxious thermal and chemical responses, and its overactivation contributes to itch and burning sensations observed in inflammatory dermatoses [12]. CB_2_ receptors on peripheral nerve terminals exert anti-nociceptive and anti-inflammatory effects by suppressing the release of pro-inflammatory mediators from adjacent immune cells [67]. Through this neuro-immune interface, sensory nerves integrate ECS signals into pain perception, itch modulation, and cutaneous neuroinflammation.

Eccrine sweat gland epithelium expresses CB_1_, CB_2_, and ECS enzymes, supporting local endocannabinoid signaling [12,24]. AEA and 2-AG activate MAPK pathways in eccrine cells, influencing cell cycle, apoptosis, and lipid synthesis [68]. TRPV4 is consistently expressed, whereas TRPV1 exhibits functional roles in thermosensory regulation, though these are inconsistently reported [13,53,69]. Thus, ECS activity may modulate both sweat secretion and its composition, impacting hydration and the skin microbiome.

Hair-follicle epithelium expresses CB_1_/CB_2_ and TRPV1/3/4. CB_1_ signaling suppresses mast cell activation in the connective sheath, reducing perifollicular inflammation [63,64]. TRPV3/4 regulate junctional integrity, barrier repair, and hair-cycle dynamics [70].

Sebocytes produce AEA and 2-AG and predominantly express CB_2_, which drives lipogenesis and apoptosis in an autocrine/paracrine loop [13,37]. They express both synthetic (NAPE-PLD, DAGLβ) and degradative (FAAH, MAGL) enzymes. Targeting this signaling axis holds therapeutic potential in acne vulgaris, where excessive lipogenesis and inflammation contribute to the disease’s pathology [37,71].

Melanocytes express CB_1_, CB_2_, and TRPV1. AEA elicits biphasic, dose-dependent effects on melanogenesis and survival [72]. Although FAAH is present, endogenous eCB production is uncertain. GPR55 is upregulated in melanoma and squamous cell carcinoma, implicating it in the pathology of pigment cells [58].

Dermal fibroblasts express CB_1_, CB_2_, TRPV4, and FAAH [53]. Early CB_2_ activation after injury reduces the release of pro-inflammatory mediators, modulates fibroblast-to-myofibroblast differentiation, and promotes re-epithelialization. TRPV4 and Transient receptor potential canonical 6 (TRPC6) mediate mechanotransduction, influencing contractility and extracellular matrix remodeling [73]. While GPR55 expression has not been confirmed in human dermal fibroblasts, its established roles in other mesenchymal cells suggest potential involvement in migration and matrix regulation [74].

Vascular endothelial cells express CB_1_, enabling cannabinoids to modulate vasomotor tone [75]. CB_2_ in perivascular immune cells regulates leukocyte adhesion and extravasation, contributing to vascular immune homeostasis. TRPV4 acts as a shear stress sensor, influencing permeability and mechanosensory responses [76]. FAAH expression is variable, suggesting local modulation of AEA availability. While GPR55 localization in dermal vessels has not been confirmed, its vascular roles in angiogenesis and migration imply potential involvement.

In summary, the distributed expression of CB_1_, CB_2_, TRP channels, GPR55, and their associated enzymes across cutaneous compartments underscores the ECS as a unifying regulator of skin homeostasis. Dysregulation of these pathways contributes to inflammatory, neoplastic, and degenerative dermatological disorders, positioning the ECS as a compelling therapeutic target.

## 3. Dysregulation of the Cutaneous Endocannabinoid System in Dermatological Disorders

The cutaneous ECS is a dynamic regulatory network that maintains skin homeostasis by coordinating barrier integrity, immune tone, inflammation, nociception, and adnexal activity. In diverse dermatological disorders, including atopic dermatitis, psoriasis, acne vulgaris, and chronic wounds, ECS signaling becomes disrupted through altered receptor expression, imbalanced ligand levels, and dysregulated enzyme activity. Because each of these conditions has distinct but overlapping pathogenic mechanisms, it is helpful to consider how their physiology intersects with ECS dysfunction. These molecular shifts often correlate with disease severity and clinical phenotype [13,14,15].

Atopic dermatitis is characterized by epidermal barrier impairment, with reduced filaggrin (FLG) and ceramide content and increased transepidermal water loss, which facilitates allergen entry and microbial colonization. Immunologically, the disease is driven by T-helper type 2 (Th2) cytokines, notably IL-4 and IL-13, which induce keratinocyte-derived alarmins such as TSLP, thereby perpetuating inflammation and pruritus [77,78]. In lesional skin, AEA and 2-AG levels are reduced, while FAAH activity is increased, thereby accelerating the degradation of eCBs [12]. In murine models of allergic contact dermatitis, the expression of CB_1_ and CB_2_ is diminished in keratinocytes and dermal immune cells, weakening anti-inflammatory and barrier-supportive signaling [79]. In parallel, persistent TRPV1 activation by AEA contributes to maladaptive neuroimmune signaling and itch [12]. Experimental models show that CB_2_ agonism suppresses allergic inflammation and pruritus, whereas CB_2_ deficiency exacerbates disease severity [75,80].

Psoriasis is an immune-mediated disorder driven by the IL-23/T-helper 17 (Th17) axis. Interleukin-17 (IL-17) and interleukin-22 (IL-22) promote keratinocyte hyperproliferation and aberrant differentiation, while dendritic cell-derived TNF-α and IL-23 sustain chronic T-cell activation, neutrophil infiltration, and angiogenesis [81,82]. In human keratinocytes, phytocannabinoids such as CBD and cannabigerol repress differentiation genes through epigenetic mechanisms, with CBD exerting CB_1_-dependent effects [72]. In contrast, CB_2_ deficiency exacerbates imiquimod-induced psoriasiform dermatitis, characterized by elevated TNF-α, IL-1β, and IL-17 expression, which is reversed by CB_2_ activation [83]. Moreover, in differentiating keratinocytes, increased FAAH activity reduces AEA levels and, through CB_1_ signaling, impairs epidermal differentiation programs [84]. Collectively, these alterations disrupt keratinocyte–immune crosstalk and amplify the IL-23/Th17 axis, thereby sustaining chronic psoriatic inflammation.

Acne arises from a combination of sebaceous gland hyperactivity, follicular hyperkeratinization, and microbial colonization by *Cutibacterium acnes*, which generates pro-inflammatory mediators such as IL-1β. Androgen-driven sebocyte lipogenesis further amplifies this inflammatory loop [85,86]. Human sebocytes express CB_2_ and actively produce eCBs; CB_2_–MAPK signaling drives lipogenesis and apoptosis, establishing an autocrine/paracrine loop that regulates sebum output [37]. Dysregulation, particularly increased MAGL activity, which lowers 2-AG tone, favors hyperseborrhoea and pro-inflammatory signaling. Moreover, the modulation of eCBs on transporters and enzymes alters lipid production and cytokine release (IL-6, IL-8), thereby amplifying responses to *Cutibacterium acnes* [71]. Collectively, ECS imbalance in sebocytes contributes to both the seborrhoeic and inflammatory features of acne.

Normal wound healing requires a coordinated transition from inflammation to proliferation. In chronic wounds, macrophages persist in an M1 pro-inflammatory state, angiogenesis is impaired, and fibroblast differentiation into myofibroblasts is delayed, resulting in a stalled wound repair process. Oxidative stress and protease excess further degrade the extracellular matrix and growth factors [87,88]. ECS inputs influence each of these processes. In incisional-wound models, CB_2_ agonists accelerate closure by suppressing M1 macrophage programs and reducing pro-inflammatory cytokines, thereby promoting re-epithelialization [14]. ECS inputs also influence angiogenesis, fibroblast-to-myofibroblast differentiation, and extracellular matrix remodeling, all of which are critical to wound healing. Reduced CB_2_ tone therefore impairs the inflammatory-to-repair transition, predisposing to delayed healing and chronicity.

Taken together, dysregulation of the cutaneous ECS, through diminished receptor activity, excessive eCB degradation, and amplified inflammatory signaling, represents a shared pathogenic feature across major dermatologic diseases. This provides a conceptual bridge toward receptor-selective therapeutic strategies discussed in the next section.

## 4. Rationale for CB_2_ Targeting in Dermatology

Across atopic dermatitis, psoriasis, acne, and chronic wounds, a convergent pattern emerges: diminished CB_2_ signaling, accelerated degradation of eCBs, heightened cytokine activity, and persistent immune and sensory activation. Collectively, these alterations position CB_2_ as a central regulatory node within the cutaneous ECS, capable of modulating inflammation, pruritus, barrier dysfunction, and tissue repair, thereby providing a mechanistic rationale for therapeutic strategies that selectively enhance CB_2_ activity [13,15]. Reflecting these mechanistic links, cannabinoid-based approaches have already been explored in dermatology, with reviews highlighting the therapeutic potential of CBD and related compounds in conditions such as atopic dermatitis, psoriasis, acne, pruritus, and wound healing. Early preclinical and human studies report improvements in inflammation, pruritus, and barrier function [13,15,55,56,89,90]. Collectively, this growing body of evidence underscores cannabinoids as a feasible therapeutic class for skin disorders and strengthens the case for developing more selective, peripherally active CB_2_ ligands.

The CB_2_ receptors are enriched on peripheral immune cells and expressed by keratinocytes, fibroblasts, sebocytes, and small-diameter sensory fibers, while exhibiting minimal CNS distribution [15]. This peripheral bias offers a dermatologic advantage, providing selective modulation of inflammation and pruritus without the psychotropic liability associated with CB_1_ activation [91,92].

Mechanistically, CB_2_ signaling suppresses nuclear factor kappa B (NF-κB)/MAPK pathways, thereby reducing pro-inflammatory mediators including TNF-α, IL-1β, and Interleukin-6 (IL-6). It limits leukocyte recruitment, promotes resolution-phase macrophage programs, and reduces nociceptor excitability. Cross-talk with TRP channels provides additional levers for regulating itch and pain [93]. In wound models, CB_2_ agonism accelerates re-epithelialization and improves the inflammatory milieu, thereby facilitating repair [14].

Significantly, dysregulated CB_2_ tone, characterized by reduced receptor activity, increased eCBs catabolism, and heightened cytokine release, has been documented in atopic dermatitis, psoriasis, acne, and chronic wounds. These findings underscore CB_2_ as a coherent nodal target across inflammatory and barrier-disrupted dermatoses. While most evidence supports the anti-inflammatory and pro-repair benefits, cell-specific responses (e.g., in sebocytes or specific dermatitis models) suggest that outcomes may vary depending on the disease context, which should inform translational strategies [80,94]. Because signaling is largely peripheral, topical CB_2_-selective ligands can concentrate benefits in the skin while minimizing systemic exposure, offering steroid-sparing, antipruritic, and pro-repair potential in eczematous disorders, photo- or irritant-induced inflammation, and wounds. Among candidate ligands, BCP stands out as a natural, highly selective CB_2_ agonist with favorable developability, provided oxidation is controlled, making it the most compelling lead for topical dermatologic applications [21,39].

## 5. β-Caryophyllene: Chemistry and Developability for Topical Use

BCP is a bicyclic sesquiterpene present in the essential oils of Piper nigrum (black pepper), clove, oregano, basil, rosemary, hops, copaiba balsam, cinnamon, and Cannabis sativa [23,95,96]. Structurally, BCP (C_15_H_24_; molecular weight = 204.35 g·mol^−1^) contains a fused cyclobutane–cyclohexene scaffold with an exocyclic double bond [97]. This rigid, hydrophobic framework underlies its strong affinity for the CB_2_ receptor while conferring negligible CB_1_ activity, resulting in the absence of psychoactive effects [98,99]. With a molecular weight below 500 Da and high lipophilicity (logP ≈ 4.5–5), BCP readily partitions into the lipid matrix of the stratum corneum, forming a local reservoir that sustains epidermal and dermal exposure [97]. These physicochemical characteristics satisfy several criteria for topical and transdermal delivery. In pharmaceutical development, supercritical CO_2_ extraction is generally preferred over steam or hydrodistillation because it avoids the use of organic solvents and preserves thermolabile constituents [100].

**Figure 2:**
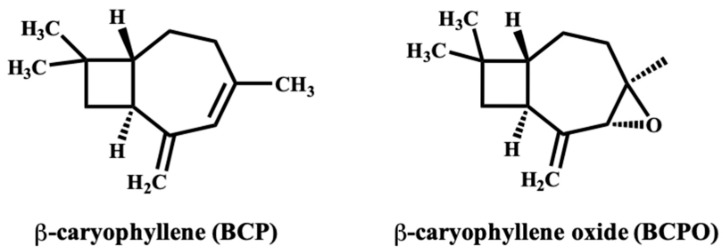
Structures of β-caryophyllene (BCP) and β-caryophyllene oxide (BCPO). Figure created with ChemDraw v25.0.

Despite these advantages, BCP exhibits notable chemical instability. Its exocyclic double bond is highly prone to autoxidation, producing BCPO, a derivative with distinct pharmacological properties and an increased risk of sensitization (Figure 2) [40]. Oxidative degradation and volatility during manufacturing and storage can compromise potency, reproducibility, and safety. Therefore, production and packaging should minimize oxygen exposure, employ inert atmospheres or antioxidant excipients, and monitor peroxide values and the BCP: BCPO ratio at batch release [23]. BCP’s volatility and hydrophobicity also pose formulation challenges. Evaporative loss from ethanol-based or other volatile vehicles can reduce the effective dose and alter dermal penetration. At the same time, variability in excipient composition or solvent polarity may influence solubility, stability, and release kinetics. Standardization of vehicle composition and stability-indicating assays is therefore critical to ensure consistent performance across formulations.

Encapsulation technologies have been developed to overcome these limitations. Incorporating BCP into nanoemulsions, liposomes, solid-lipid nanoparticles, or polymeric carriers protects against oxidation, reduces volatility, and enables controlled release, thereby improving dermal bioavailability [101,102,103]. Ethanol–terpene cosolvent systems and nanocolloidal formulations further enhance permeation while maintaining a predominantly local pharmacokinetic profile. Most topically applied BCP remains confined to the skin with minimal systemic exposure; however, under barrier-compromised conditions or when potent enhancers are used, limited systemic absorption may occur, followed by metabolism via CYP3A and CYP2C isoenzymes and subsequent conjugation [104]. Because of its CB_2_ selectivity, BCP avoids CB_1_-mediated psychoactive effects. The interaction potential is low, although in vitro studies indicate weak inhibition of CYP3A4 and CYP2C and possible modulation of P-glycoprotein [105,106]. Caution may therefore be warranted during occlusive or transdermal use with drugs possessing a narrow therapeutic index, such as ciclosporin or warfarin.

In summary, BCP combines natural abundance, CB_2_-selective pharmacology, and favorable dermal retention, making it an attractive candidate for dermatologic formulations. Volatility, autoxidation, and formulation variability remain key limitations but can be mitigated through encapsulation approaches and standardized manufacturing. Ultimately, successful translation will depend on whether these developability strategies yield stable formulations capable of consistent CB_2_-mediated signaling and measurable therapeutic benefit, a focus of the following section.

## 6. Molecular Pharmacology of β-Caryophyllene in Skin

BCP is a highly selective CB_2_ agonist with negligible CB_1_ activity [19]. While BCP exhibits a broad pharmacological repertoire, including anti-inflammatory, antioxidant, analgesic, and metabolic effects, its molecular actions in the skin are of relevance to dermatological applications. CB_2_ agonism remains the dominant pharmacological mechanism; however, BCP also modulates NF-κB, engages PPARγ, and interacts with TRP-linked pathways, collectively underpinning its anti-inflammatory, antioxidant, and barrier-restoring properties [69,107,108].

CB_2_ (CNR2) activation initiates canonical Gi/o coupling that inhibits adenylyl cyclase and reduces cyclic adenosine monophosphate (cAMP) levels. This downregulation decreases protein kinase A (PKA) activity and phosphorylation of cAMP response element–binding protein (CREB), a transcription factor that regulates gene expression. In parallel, CB_2_ signaling modulates mitogen-activated protein kinases (MAPKs), including extracellular signal-regulated kinase (ERK1/2), p38 MAPK, and c-Jun N-terminal kinase (JNK), and converges on nuclear factor kappa B (NF-κB), reducing the expression of cyclooxygenase-2 (COX-2), TNF-α, IL-1β, and IL-6 [22,109]. These effects collectively produce strong anti-inflammatory responses, aligning with therapeutic targets in psoriasis and atopic dermatitis.

In keratinocytes, BCP specifically counteracts Th2-driven inflammation by suppressing the IL-4/IL-13–MAPK–early growth response 1 (EGR1) axis, which otherwise promotes thymic stromal lymphopoietin (TSLP), a central epithelial alarmin in atopic dermatitis [30,110]. By dampening this upstream cascade, BCP interrupts the positive feedback loop that sustains Th2 cytokine production and barrier dysfunction. Inhibition of TSLP blunts dendritic cell activation and subsequent Th2 polarization, linking BCP to improved outcomes in allergic and eczematous disorders [111].

In parallel, BCP augments antioxidant defenses through CB_2_-mediated activation of the Akt–nuclear factor erythroid 2 2-related factor 2 (Nrf2)–antioxidant response element (ARE) pathway. Nrf2 is a transcription factor that, upon activation, translocates to the nucleus and binds specific DNA motifs known as AREs, thereby initiating the transcription of cytoprotective genes [112]. This induces heme oxygenase-1 (HO-1), increases intracellular glutathione (GSH) content, and enhances superoxide dismutase (SOD) activity, while reducing reactive oxygen species (ROS) and lipid peroxidation markers, such as malondialdehyde (MDA) [113,114]. Preclinical models confirm that BCP mitigates oxidative stress by upregulating Nrf2 targets and suppressing lipid peroxidation [34,115]. Because Nrf2 also protects against UV-induced oxidative injury in keratinocytes, this antioxidant pathway contributes to photoprotection, wound repair, and accelerated re-epithelialization [116].

Barrier restoration is another key axis of BCP action. In Th2-driven inflammation, IL-4 and IL-13 downregulate epidermal differentiation complex (EDC) proteins, including FLG, involucrin (IVL), and loricrin (LOR), via STAT6/STAT3 signaling, weakening barrier integrity and increasing transepidermal water loss (TEWL) [117,118]. Although direct evidence in keratinocytes is lacking, CB_2_ agonism is proposed to attenuate this pathway and sustain EDC protein expression. Crosstalk with PPARγ may provide an additional barrier-reinforcing mechanism, as PPARγ activation promotes lipid synthesis and keratinocyte differentiation [108]. BCP has been shown to activate PPARγ in vivo, contributing to anti-inflammatory and anti-fibrotic effects [119]. Dual CB_2_/PPARγ ligands, such as VCE-004.8, further demonstrate how cannabinoid and PPARγ signaling inhibit pro-fibrotic pathways and enhance tissue integrity [120]. In dermatology, this dual CB_2_/PPARγ axis is particularly relevant to skin repair and fibrotic dermopathies [121].

BCP also engages neurosensory pathways relevant to itch and pain modulation. CB_2_ activation in keratinocytes promotes β-endorphin release, which acts on μ-opioid receptors in sensory neurons to alleviate itch and pain [122,123,124]. Moreover, modulation of TRP channels, particularly TRPV4, links CB_2_ signaling to antipruritic and antinociceptive outcomes [33]. Other TRPs (TRPV1, TRPV3, TRPA1) have also been implicated in itch and inflammation, suggesting possible BCP/TRP crosstalk; however, the evidence remains preliminary.

**Figure 3:**
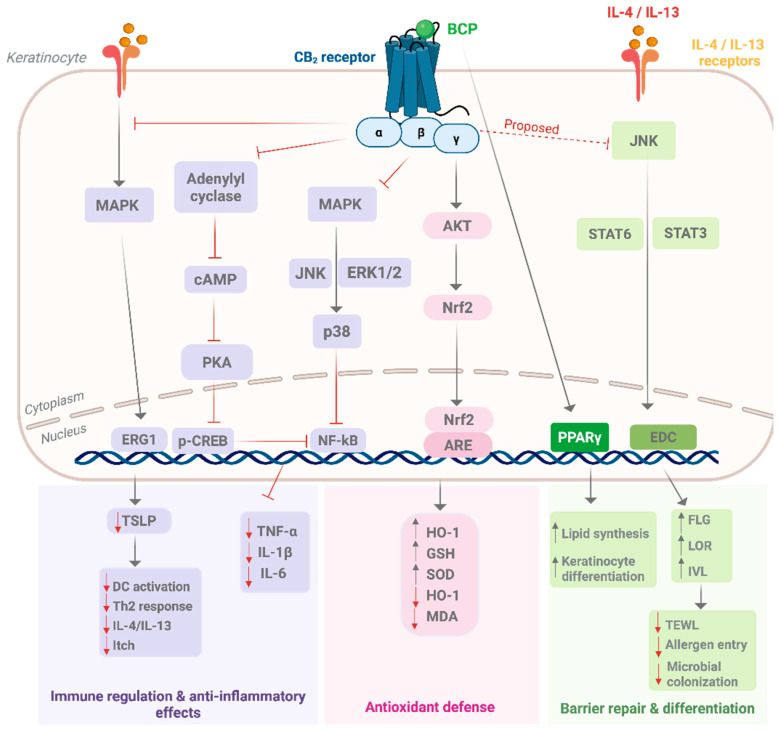
Molecular pathways engaged by β-caryophyllene in keratinocytes relevant to dermatologic disorders. BCP activates CB_2_ receptors to regulate immune responses, enhance antioxidant defense, and restore epidermal barrier function. Grey arrows indicate activation, red T-ended lines indicate inhibition, and dashed red lines denote potential but unconfirmed inhibitory effects on IL-4/IL-13–STAT6/STAT3 signaling. Arrow notation: solid line = stimulation/activation; T-bar = inhibition. Abbreviations: AKT, protein kinase B; cAMP, cyclic adenosine monophosphate; CB_2_, cannabinoid receptor type 2; DC, dendritic cell; EDC, epidermal differentiation complex; EGR1, Early Growth Response 1; ERK, extracellular signal–regulated kinase; FLG, filaggrin; GSH, glutathione; HO-1, heme oxygenase-1; IL, interleukin; IVL, involucrin; JNK, c-Jun N-terminal kinase; LOR, loricrin; MAPK, mitogen-activated protein kinase; MDA, malondialdehyde; NF-κB, nuclear factor kappa-light-chain-enhancer of activated B cells; Nrf2, nuclear factor erythroid 2–related factor 2; PKA, protein kinase A; p-CREB, phosphorylated cAMP response element-binding protein; PPARγ, peroxisome proliferator–activated receptor gamma; SOD, superoxide dismutase; STAT, signal transducer and activator of transcription; TEWL, transepidermal water loss; TSLP, thymic stromal lymphopoietin. Created in BioRender. Bagher, A. (2025) https://BioRender.com/iahviub.

Collectively, these immune, barrier, antioxidant, and neurosensory mechanisms position BCP as a multifunctional modulator within the cutaneous endocannabinoid system. Its integrated actions map directly onto primary dermatological conditions, atopic dermatitis (Th2/TSLP blockade), psoriasis (NF-κB suppression), photoaging and wound repair (Nrf2 activation), fibrosis and barrier loss (PPARγ signaling), and pruritus (opioid/TRP pathways). Figure 3 summarizes these mechanisms, illustrating how BCP may restore cutaneous homeostasis through coordinated regulation of inflammation, oxidative stress, barrier function, and sensory signaling.

## 7. Preclinical and Clinical Evidence of β-Caryophyllene in Skin Disorders

Numerous studies have examined the therapeutic potential of BCP in the treatment of cutaneous diseases. We integrate in vitro (cell and ex vivo), in vivo topical, and human data across inflammation, barrier/differentiation, wound repair, and pruritus, mapping results to the mechanistic framework described above. Because many reports use BCP-rich botanicals (e.g., copaiba, clove), we separate purified BCP from mixtures to avoid misattribution and interpret signals in the context of formulation, concentration, exposure time, route, and study quality.

### 7.1. Mini-Methods (Search, Eligibility, and Appraisal)

A structured literature search was conducted in PubMed, Embase, and Web of Science from database inception to 30 July 2025, in accordance with PRISMA 2020 guidelines for transparent reporting of systematic reviews. The Boolean search strategy combined keywords and MeSH terms as follows: (“β-caryophyllene” OR “BCP”) AND (skin OR keratinocyte OR fibroblast OR sebocyte OR “wound healing” OR dermatitis OR psoriasis OR burn OR pruritus) AND (topical OR cutaneous). The initial broad search without the topical filter returned 315 records in PubMed. Applying the topical/dermal filter reduced the yield to 37 records. The same search syntax was used in Embase and Web of Science, and all results were cross-referenced to remove duplicates, yielding 64 unique articles for screening.

Studies were included if they investigated purified BCP (≥95% purity confirmed by GC-MS or HPLC) or analytically characterized BCP-dominant extracts applied to cutaneous cells, ex vivo skin, in vivo topical models, or human participants. Studies involving systemic or oral administration without cutaneous endpoints, unquantified mixtures, review articles, conference abstracts, or non-English publications were excluded [125,126]. No clinical studies using purified BCP were identified; therefore, BCP-rich multicomponent botanical formulations were included for contextual comparison and to provide indirect clinical insight.

Eligible studies evaluated one or more of the following outcomes: modulation of inflammatory signaling (NF-κB, MAPK, cytokine profiles), epidermal differentiation or barrier integrity (FLG, IVL, LOR, TEER), wound healing or re-epithelialization rate, pruritus behavior, oxidative-stress parameters, and local or systemic safety endpoints.

Two independent reviewers screened titles, abstracts, and full texts, and extracted data using predefined templates. Discrepancies were resolved by consensus. Extracted variables included experimental model, species or cell line, formulation type, and vehicle, BCP concentration, exposure duration, occlusion conditions, formulation stability, and normalized dermal dose (µg·cm^−2^).

Preclinical animal studies were evaluated according to the ARRIVE 2.0 guidelines [127], while clinical studies were appraised using the Risk of Bias 2 (RoB 2) tool, Version 2.0 [128]. For in vitro models, primary keratinocytes or N/TERT cell lines were prioritized over immortalized HaCaT cells when assessing barrier-related outcomes.

Because of substantial heterogeneity in study design, formulations, and dosing regimens, meta-analysis was not feasible. Data were synthesized qualitatively, emphasizing the direction and magnitude of observed effects and mechanistic concordance across models. Methodological considerations included vehicle or DMSO variability, oxidative stability of BCP, and verification of receptor expression [39,103]. Publication bias was not formally assessed due to heterogeneity and limited sample size. Eligible preclinical studies are summarized in Table 1.

### 7.2. Preclinical Evidence

#### 7.2.1. In Vitro Studies

Most keratinocyte and fibroblast assays tested purified BCP at concentrations of 0.5–10 µM for 24–48 h, with minimal cytotoxicity. CB_2_ involvement was frequently confirmed through reversal by antagonists such as AM630 or SR144528. However, methodological heterogeneity, including vehicle effects, weak oxidation controls, and reliance on immortalized HaCaT keratinocytes, limits the generalizability of the results. Greater weight is therefore placed on studies that used receptor controls, primary keratinocytes, or explicit oxidation analytics.

Across keratinocyte models, BCP consistently demonstrates anti-inflammatory activity. In LPS-challenged HaCaT cells, BCP (1–10 µM; 24 h) suppressed NF-κB p65 phosphorylation, COX-2, and IL-1β, with these effects reversed by AM630, confirming CB_2_ dependence [129]. In IL-4-stimulated keratinocytes, which mimic Th2-driven inflammation in atopic dermatitis, brief submicromolar pretreatment reduced TSLP expression at both mRNA and protein levels [30]. Despite these findings, HaCaT cells exhibit low transepithelial electrical resistance (TEER) and weak barrier properties. Confirmation in primary keratinocytes or N/TERT2G lines, with readouts such as differentiation markers (FLG, IVL, LOR) and TEER, is still required [130]. The suppression of NF-κB and IL-1β also overlaps with the pathogenic drivers of psoriasis, suggesting that BCP’s keratinocyte effects may extend to multiple chronic inflammatory dermatoses.

In dermal fibroblasts, BCP has been shown to promote migration and repair-associated signaling, while reducing the release of inflammatory mediators. Scratch and Boyden chamber assays generally report enhanced migration and reduced cytokine output, although proliferation is not always clearly distinguished from migration. Extracellular matrix outcomes, such as the collagen I/III ratio and MMP/TIMP balance, remain inconsistently assessed [131]. CB_2_ involvement has been rarely investigated using antagonists or siRNA approaches, resulting in limited mechanistic understanding. Nevertheless, evidence supports a pro-repair role for BCP, with potential relevance for wound healing, chronic ulcers, and psoriatic dermal remodeling, where cytokine imbalance and extracellular matrix dysregulation are central.

Evidence in sebocytes remains limited. SZ95 sebocytes are CB_2_-positive and maintain an intrinsic endocannabinoid tone that regulates lipogenesis and apoptosis, providing a biological basis for BCP’s influence on lipid homeostasis [13,37].

Translationally, CB_2_ agonism may promote neutral lipid accumulation, while anti-inflammatory activity could mitigate cytokine-driven sebogenesis [71,132]. However, purified BCP has not been systematically studied under exposure to *Cutibacterium acnes* or cytokine challenge. Key gaps include quantitative lipidomics (e.g., Nile Red staining), assessment of squalene oxidation, and cytokine profiling (IL-1β, IL-6, TNF-α) with CB_2_ antagonism or siRNA confirmation. Without such studies, sebocyte-specific effects of BCP remain plausible but unproven [12,37].

Collectively, in vitro data support the CB_2_-mediated anti-inflammatory and barrier-restorative effects in keratinocytes, pro-migratory signals in fibroblasts, and a possible, but as yet unconfirmed, role in sebocyte regulation. These findings provide the mechanistic backdrop for evaluating BCP in in vivo topical models and early human studies, which are addressed in the following subsections.

#### 7.2.2. In Vivo Animal Models

Reporting standards across topical in vivo studies are heterogeneous, with randomization and blinding often absent, active comparators uncommon, and dermal pharmacokinetics (PK) rarely assessed. Bioactive vehicles, such as olive oil or copaiba oleoresin, were frequently used; therefore, greater weight is given to studies employing purified BCP or analytically verified fractions delivered in inert carriers. Despite these limitations, convergent findings emerge across wound-healing, atopic dermatitis, and irritant models.

In a BALB/c dermatitis model, topical BCP (0.001–100 µg/mL, 20 days) improved AD-like lesions, reducing epidermal and dermal thickening, limiting inflammatory infiltration (including mast cells), and suppressing keratinocyte EGR1 and TSLP, consistent with IL-4/MAPK/EGR1/TSLP signaling [30]. In contrast, higher concentrations (0.1–10 mg/mL, repeated epicutaneous dosing) induced dose-dependent pruritic dermatitis with scratching, mast cell recruitment, reduced FLG, and elevated serum IgE. CBD did not reproduce these effects [36]. Together, these studies suggest an exposure-dependent therapeutic window: BCP is anti-inflammatory at treatment-range doses but sensitizing with high-dose, repeated exposure, highlighting the importance of formulation control, oxidation monitoring, and CB_2_ verification.

In rodent excision models, topical BCP (50 mg/kg in olive oil, ~50 µL/wound, daily) accelerated wound repair. Wounds in mice closed earlier with BCP under a ring-reservoir system; this effect was reduced by AM630, confirming CB_2_ involvement, and was more pronounced in females. Mechanistic analyses revealed enhanced keratinocyte proliferation and migration, activation of hair-follicle bulge, and upregulation of TRPV4, alongside suppression of IL-1β/IL-6 and induction of Hedgehog and Wnt/β-catenin signaling [33]. In rats, daily application of 1% *w*/*w* BCP emulgel improved closure and remodeling, with up-regulation of laminin-γ2, desmoglein-3, and collagen, reduced pro-inflammatory cytokines (TNF-α, IFN-γ, IL-1β, IL-6), increased IL-10, and antioxidant activity (elevated glutathione peroxidase, GPx), without hepatic or renal toxicity compared with comparators [32]. Collectively, wound-healing studies indicate a consistent pro-regenerative, anti-inflammatory, and antioxidant signal; however, interpretation is tempered by vehicle bioactivity, unmeasured dermal exposure, and the lack of dose normalization.

Most evidence in the burn, UVR, and irritant domain derives from BCP-rich botanicals (e.g., copaiba oleoresin) rather than purified BCP, limiting causal attribution. In a UVB sunburn mouse model (0.75 J·cm^−2^), a 3% copaiba cream-GC-MS verified to contain BCP among major sesquiterpenes-attenuated mechanical allodynia (≈65% inhibition on day 2) and abolished thermal hyperalgesia, with reduced leukocyte infiltration but unchanged dermal thickness; the formulation remained stable for ~2 months [35]. Pruritic outcomes appear receptor- and dose-dependent: the non-selective agonist WIN55,212-2 reduced experimentally induced scratching via CB_2_ (blocked by SR144528 or CB_2_ knockout), whereas high-dose topical BCP induced dermatitis-like changes with pruritus in mice [36,133]. Together, these findings warrant cautious optimism but emphasize the need for purified BCP tested at dermally relevant exposures, with receptor controls and photostability analytics, to support causal inference and translational credibility.

In summary, preclinical evidence supports BCP as a pro-regenerative and anti-inflammatory agent, with additional antioxidant and analgesic properties. However, translation is constrained by dose, vehicle, and stability issues, underscoring the need for purified BCP tested at dermally relevant exposures, with receptor controls and photostability analytics, before advancing to human studies.

### 7.3. Clinical Evidence

Building on the encouraging preclinical evidence, peer-reviewed efficacy data for isolated topical BCP in dermatologic applications are currently unavailable. All existing human data derive from BCP-rich but multicomponent botanical systems, most notably Copaifera oleoresins, in which BCP is the predominant, yet not exclusive, sesquiterpene constituent. Consequently, these findings should be interpreted as indirect evidence supporting BCP’s potential clinical relevance rather than as compound-specific proof of efficacy [134].

The most substantial clinical data come from two randomized controlled trials evaluating copaiba-based formulations. Waibel et al. (2021) [135] conducted a prospective, randomized, double-blind, placebo-controlled study in adults with abnormal scars, evaluating a silicone gel containing copaiba oil. The intervention demonstrated a significant improvement in scores on the Manchester Scar Scale, a validated clinical tool for assessing scar quality and severity, compared to the placebo over 84 days [135]. Cardinelli et al. (2024) [136] conducted a three-arm, randomized, double-blind clinical trial in patients with skin tears, comparing polymeric hydrogels containing 2% or 10% *Copaifera multijuga* oil with a vehicle hydrogel. All wounds healed, with the 2% hydrogel accelerating closure, and no local or systemic adverse events were reported [136]. In both studies, however, the investigational products were multicomponent oleoresins containing numerous terpenoids and acids, precluding definitive attribution of the observed effects to BCP itself.

Beyond these randomized trials, two smaller clinical studies provide non-definitive supportive evidence for BCP-rich copaiba derivatives. Leite et al. (2023) [137] described a case series of patients with chronic wounds treated with topical copaiba oleoresin, reporting progressive wound closure over variable treatment durations [137]. In acne vulgaris, da Silva et al. (2019) [138] conducted a double-blind, placebo-controlled trial of *Copaifera langsdorffii* essential oil, demonstrating clinical improvement in acne severity compared with placebo [138]. While encouraging, both interventions employed multicomponent botanical preparations rather than purified BCP, and neither incorporated on-skin pharmacokinetic or oxidation monitoring, limiting mechanistic attribution and reinforcing the need for vehicle-controlled, dose-ranging trials of purified BCP.

Taken together, available human studies provide encouraging yet indirect clinical signals from BCP-rich *Copaifera* preparations, notably in wound repair and scar remodeling. Nonetheless, the absence of purified BCP trials and the lack of formulation-specific pharmacokinetic or stability data preclude definitive conclusions regarding BCP’s dermatologic efficacy. To generate drug-quality evidence, future investigations should employ chemically defined, oxidation-controlled formulations that can sustain dermal exposure under finite-dose, open-system conditions. These clinical investigations are summarized in Table 2, and Section 8 outlines formulation and packaging strategies designed to meet these translational requirements for topical BCP.

## 8. Formulation Strategies for Topical β-Caryophyllene

A topical BCP product must achieve high dermal levels with minimal barrier disruption while limiting evaporation and oxidative degradation. An approach to stability-by-design that integrates antioxidants, peroxide monitoring, and oxygen- and light-barrier packaging is essential. Quantification of both BCP and its oxidation product, BCPO, combined with volatility and permeation studies, such as headspace Gas Chromatography (GC)**,** IVRT, and IVPT, provides a minimal analytical framework to ensure formulation reliability [32,39,139]. To enhance reproducibility and translational relevance, we outline minimum reporting standards for topical BCP formulations (Table 3) [140,141]. Complementing this, key experimental evidence from delivery and targeting systems is summarized in Table 4, which illustrates how formulation choices impact dermal exposure, PD, and stability.

Formulation evidence spans from relatively simple semisolid systems to more complex nanocarriers. Among semisolids, Gushiken et al. (2022) reported that a 1% *w*/*w* BCP emulgel (medium-chain triglyceride oil + propylene glycol; polymeric emulsifier) accelerated wound closure and shifted cytokine/antioxidant markers toward healing in a rat excision-wound model [32]. However, the formulation did not incorporate antioxidant systems or monitoring of BCPO, which limits the interpretation of its stability and reproducibility. These findings nonetheless indicate that relatively simple semisolid systems can provide PD benefit when a suitable oil phase and cosolvent maintain BCP in a dispersed state [32]. Future studies should incorporate safeguards against oxidation and validated analytical controls to support clinical translation.

Compared with conventional semisolids, nanocolloidal systems—including microemulsion hydrogels and nanoemulgels—provide stronger evidence for the link between formulation design, dermal exposure, and PD outcomes. Alharthi et al. (2023) reported that a 1% BCP microemulsion-type gel (sub-100 nm, >87% entrapment) nearly doubled ex vivo skin flux versus a conventional gel and enhanced anti-inflammatory efficacy [142]. Weimer et al. (2022) [103] found that BCPO exhibited distinct dermal PK and PD profiles, emphasizing the need to analyze each sesquiterpene separately [103]. Overall, nanocolloids improve dermal BCP exposure, and efficacy correlates with measured skin levels rather than droplet size or carrier composition. However, antioxidant inclusion and BCPO quantification remain inconsistently reported, leaving oxidative stability underexplored.

Nanostructured lipid carriers (NLCs) further illustrate the formulation–performance relationship. Ghazwani et al. (2023) [143] showed that BCP-loaded NLCs (~211 nm, ~87% entrapment) exhibited 24 h controlled release and ~1.9-fold higher ex vivo skin retention than a conventional gel [143]. These lipid reservoirs help buffer volatility and prolong cutaneous residence. Nevertheless, antioxidant strategies, BCPO stability data, and in vivo safety assessments (e.g., HRIPT) are still lacking. Vesicular systems (liposomes, ethosomes, transfersomes) remain less developed; a redispersible liposomal BCP powder improved manufacturability but has not been evaluated for dermal PK/PD [144]. Thus, nanoemulsions and NLCs currently represent the most promising yet incompletely validated BCP vehicles.

BCP also demonstrates potential as a penetration co-enhancer. Tang et al. (2023) showed that BCP modestly increased stratum-corneum lipid fluidity and potentiated 5-aminolevulinic acid photodynamic therapy in mice [145]. Although studied in a different indication, this mechanistic signal supports BCP’s ability to transiently loosen skin lipids and enhance the uptake of co-administered actives. To avoid redundancy, all discussion of BCP’s co-enhancer activity has been consolidated here. Future work should confirm this enhancer role under finite-dose, open-system conditions, with transepidermal water loss (TEWL) and erythema monitoring and explicit BCPO quantification.

Beyond BCP-specific data, general penetration strategies remain relevant but need validation in BCP formulations. Practical measures include using low-level cosolvents with film formers or mild occlusion, minimal-effective fatty esters (e.g., isopropyl myristate) for partitioning, and carriers of ~100–300 nm for follicular targeting [146,147]. Because BCP is volatile and oxidizable, formulations should incorporate headspace-verified evaporation controls, BCPO co-assay, antioxidants under low-oxygen processing, and oxygen- and light-barrier packaging [38]. Declaring skin integrity and reporting dose/area, occlusion, temperature, and receptor-phase composition in IVPT are essential for cross-study comparison.

Co-formulating BCP with other actives can broaden efficacy and reduce required doses. With non-steroidal anti-inflammatory drugs (NSAIDs), nanoemulsified BCP plus indomethacin showed additive anti-inflammatory effects in LPS-stimulated macrophages. Still, it did not outperform free drugs in a croton oil-induced ear edema model, underscoring the need for ratio optimization and dermal exposure verification via IVPT [103,148]. With cannabinoids, low-dose CBD plus BCP additively suppressed IL-1β, IL-6, TNF-α, COX-2, and p-NF-κB in HaCaT keratinocytes through complementary activation of PPARγ and CB_2_ receptors, a mechanism relevant to pruritic inflammatory dermatoses [129]. With antimicrobials, BCP alone shows weak anti-staphylococcal activity but markedly potentiates β-lactams against Staphylococcus aureus (≈4-fold in MRSA and up to ~267-fold in fusidic acid–resistant strains), supporting its evaluation alongside topical agents such as mupirocin or fusidic acid [149]. Collectively, these findings suggest that BCP acts both as a pharmacologically active compound and as a dermal penetration co-enhancer when properly stabilized.

BCP holds substantial promise as an adjunct active in topical dermatology, combining therapeutic efficacy with formulation-enhancing properties. However, its volatility, susceptibility to oxidation, and limited PK characterization remain key challenges. Future formulation development should prioritize antioxidant protection, stability monitoring, and validated dermal PK assessment to enable consistent clinical translation and regulatory confidence.

## 9. Commercial Landscape and Patent Activity

Despite strong preclinical and mechanistic evidence, commercial use of BCP in dermatology remains limited and poorly standardized. In current markets, BCP most often appears in the International Nomenclature of Cosmetic Ingredients (INCI) as a perfuming or skin-conditioning agent, consistent with its EU CosIng classification [150]. In the United States, cosmetics are regulated but not pre-approved, and BCP is typically listed as an excipient rather than a recognized active ingredient. In dermatology-labeled products such as acne cleansers or leave-ons, therapeutic claims are anchored to approved OTC actives, while “β-caryophyllene/caryophyllene,” if disclosed, is listed as an excipient. Rinse-off formulations further reduce residence time and pharmacological plausibility [151,152]. Representative marketed cosmetic products listing BCP on the INCI label are summarized in Table 5.

High-purity BCP (≈85–95%) is widely sold as a fragrance raw material; however, retail labels rarely disclose the concentration or oxidation state (e.g., peroxide values), thereby precluding any link to pharmacological outcomes or safety. As of August 2025, no FDA-approved or European Medicines Agency (EMA)-authorized dermatology medicine lists BCP as an active ingredient. Clinical exploration remains investigational, as exemplified by a registered split-scar trial testing 20% topical BCP alone or in combination with capsicum oleoresin [153].

By contrast, the patent landscape positions BCP as a pharmacologically active therapeutic. Several international applications (e.g., US20180042890A1, WO2017190249A1) disclose topical formulations enriched with BCP, either alone or in combination with cannabinoids and terpenes, for the treatment of psoriasis, eczema, acne, seborrhea, pruritus, pain, and wound healing [154,155]. Unlike cosmetic exemplars, these patent-protected formulations specify dose-titrated concentrations and advanced carriers, such as liposomes, pluronic–lecithin organogels, and nanoemulsions, to stabilize BCP and enhance dermal penetration [156,157]. Clinical and translational syntheses echo this high-load approach, with examples citing BCP concentrations of ~153 mg/mL in multicomponent topicals [158]. While Table 5 illustrates current cosmetic positioning, the patent literature instead highlights BCP’s therapeutic potential—emphasizing its formulation as an active pharmaceutical ingredient rather than a cosmetic excipient. divergence between the cosmetic excipient status and the patent-protected therapeutic intent highlights a translational gap: BCP remains underexploited in regulated dermatology settings, despite extensive intellectual property protection. The future alignment of patent strategies with regulatory frameworks will determine whether BCP transitions from a cosmetic excipient to a validated dermatological active.

## 10. Safety, Tolerability, and Regulatory Considerations

The dermal safety of BCP has been extensively assessed by the Research Institute for Fragrance Materials (RIFM). At fragrance-use levels, it is non-genotoxic, has margins of exposure >100 for repeated-dose and fertility endpoints, and exhibits no phototoxicity, photoallergy, or sensitization when oxidation is controlled [39]. HRIPT and maximization tests further support tolerability [159,160]. The key liability is autoxidation: air exposure generates BCPO, a stronger sensitizer than native BCP, although weaker than oxidized limonene or linalool [40,161]. Preclinical studies reinforce this dose–oxidation window, with oxidized BCP provoking dermatitis-like changes in mice [36], while a 1% BCP emulgel enhanced wound healing in rats without systemic toxicity [32]. Because sensitization risk is directly linked to oxidation, preventive formulation measures remain critical. These include peroxide monitoring, stability-indicating assays that quantify both BCP and BCPO, and low-oxygen, low-light processing paired with oxygen- and UV-barrier packaging. Airless dispensers or laminated tubes are preferred, and shelf-life and in-use stability should confirm minimal BCPO formation under real-world storage.

Systemic absorption under finite-dose, open-system use on intact skin is expected to be minimal but may increase with occlusion, barrier disruption, or the use of aggressive penetration enhancers. While BCP and related congeners can inhibit CYP3A and interact with P-glycoprotein at micromolar levels in vitro, clinically meaningful drug–drug interactions remain unlikely when topical doses limit systemic uptake [105,106].

Regulatory and analytical descriptions have been streamlined here to emphasize translational aspects. BCP is recognized as GRAS for oral use (FEMA 2252), but these designations do not extend to dermatologic efficacy or drug-level safety [28,29]. In topical contexts, manufacturers must ensure ingredient safety, compliant labeling, and Good Manufacturing Practice (GMP). In contrast, clinical-grade products require validated stability assays for both BCP and BCPO, as well as documentation of oxidation control during manufacture and use. Across EU and U.S. frameworks, the unifying safety requirement is to maintain chemical stability and to define dermal exposure under intended-use conditions to establish an acceptable risk–benefit profile [162,163,164]. 

Overall, BCP remains well tolerated on the skin when oxidative conversion is prevented. Stability safeguards—antioxidant inclusion, packaging optimization, and BCPO monitoring—are essential to ensure safety and reproducibility. With these controls, BCP can progress beyond cosmetic fragrance use toward a clinically defensible dermatologic active.

## 11. Translational Outlook and Conclusions

The biological rationale for BCP in dermatology is coherent, grounded in the selective engagement of CB_2_ receptors, which leads to downstream anti-inflammatory, antipruritic, antioxidant, and barrier-supportive effects [12,21]. Preclinical studies consistently demonstrate reduced pro-inflammatory signaling, improved re-epithelialization, and attenuation of itch-related behaviors [32,33]. Nevertheless, the decisive gap is clinical: no drug-quality evidence exists with purified, dose-defined BCP, and current human signals primarily derive from multicomponent botanicals such as copaiba oleoresins, which preclude definitive attribution to BCP [135,136]. Moreover, controlled clinical trials remain scarce, formulation composition and stability vary substantially, and dermal PK data are virtually absent, hindering dose optimization and reproducibility.

Compared with existing non-steroidal topical agents such as diclofenac or pimecrolimus, BCP offers a distinct pharmacologic profile based on selective CB_2_ receptor activation, providing anti-inflammatory and barrier-supportive effects without cyclooxygenase inhibition or immunosuppressive risks [21,32,69]. Translationally, key priorities include defining standardized dermal doses through PK validation, establishing oxidation-controlled formulation specifications, and clarifying regulatory pathways distinguishing cosmetic from drug-level claims [38,39,139]. Early-phase development should focus on safety, tolerability, and proof-of-mechanism trials to position BCP among evidence-based non-steroidal dermatologic therapies [136,137].

A pragmatic translational path can be outlined. First, the administered dose must be explicitly stated and analytically verified using stability-indicating assays that track both BCP and its primary degradant, BCPO [38,39]. Second, cutaneous exposure should be measured directly; depth-resolved tape stripping with chemical analysis or in vivo confocal Raman spectroscopy can link the applied dose to skin levels [165]. Third, formulation details, including vehicle composition, antioxidants, and packaging, must be reported to ensure reproducibility, since stability directly determines efficacy and sensitization risk [38,39,139].

BCP is most likely to benefit patients with pruritic, barrier-deficient skin, such as atopic or irritant dermatitis, xerotic eczema, or post-procedure recovery [166,167]. Early clinical studies should focus on outcomes meaningful to patients, including reductions in itch intensity, improvements in dryness, redness, and sleep disturbance, time to recovery, and validated global clinical scores. Trials should be vehicle-controlled, dose-defined, and oxidation-monitored, ideally beginning with 4–8-week double-blind studies enriched for high TEWL or itch burden, with an embedded PK–PD substudy using tape-strip analytics [165].

Formulation strategies should prioritize oxidation-resistant systems that sustain dermal deposition under finite-dose, open-system use. Promising platforms include nanoemulsions, lipid carriers, and well-designed semisolids, provided they limit evaporative loss and protect against oxidation during manufacture and application [142,143,148]. Rational co-formulations, for example, with ceramide–cholesterol-free fatty acid blends for barrier repair or with PEA for itch, warrant prospective testing for additive benefits [129]. Trials should also broaden representation by including diverse phototypes and environments. Studies in the Gulf region, where darker skin tones and unique climatic factors (heat, humidity, and air conditioning) shape skin physiology, would enhance external validity and global applicability [168,169].

In conclusion, the current evidence for topical BCP remains preliminary, limited by small sample sizes, heterogeneous formulations, and the lack of pharmacokinetic validation. Nonetheless, BCP is a CB_2_-selective sesquiterpene with compelling dermatologic biology, favorable tolerability when oxidation is controlled, and extensive human exposure as a dietary and fragrance constituent. Its translation now depends on controlled, dose-defined clinical trials that measure on-skin exposure and report formulation variables transparently. If developed to drug-quality standards, BCP could advance from a cosmetic excipient to a reproducible, well-tolerated therapeutic within precision dermatology.

## Figures and Tables

**Table 1:** Summary of preclinical studies (in vitro and in vivo) on skin disorders.

Study Type/Model	Dose/Exposure	Main Outcomes	Limitations/Notes	Ref.
In vitro—Inflammation (HaCaT keratinocytes) †	1–100 µM for 24 h	↓ p65, COX-2, IL-1β at low-mid µM; no cytotoxicity ≤ 50 µM	Short exposure; no antagonist control	[129]
In vitro—Th2/TSLP axis (HaCaT keratinocytes, IL-4 challenge)	0.1–0.2 µg/mL (≈0.5–1 µM)	↓ TSLP; ↓ MAPKs; ↓ EGR1; reduced promoter activity at −369/+18	In vitro only; no animal validation	[30]
In vitro—Migration/Repair (fibroblasts, keratinocytes) *	5–10 µM for 6–24 h	↑ Migration/chemotaxis (~2× vs. control)	No mechanistic receptor analysis	[33]
In vivo—Full-thickness excisional wound (mouse; ♀ > ♂)	Topical 50 mg/kg in olive oil daily	↑ Closure; ↑ proliferation/migration markers; CB_2_ involvement	Sex difference not explained; no oxidative biomarkers	[33]
In vivo—Cutaneous wound healing (rat)	1% *w*/*w* daily	↑ Contraction/remodeling; ↓ IL-1β/IL-6/TNF-α; ↑ IL-10; no systemic toxicity	Single dose level; short follow-up	[32]
In vivo—atopic dermatitis-like model (DNCB, BALB/c mouse)	0.001–100 µg/mL; 1–2 weeks	↓ Thickening; ↓ infiltration; ↓ EGR1/TSLP	No CB_2_ blocker confirmation	[30]
In vivo (safety)—Epicutaneous sensitization (mouse)	0.1–10 mg/mL × 4 wk	Dose-dependent ↑ scratching/dermatitis; ↑ IgE; mast-cell recruitment; ↓ FLG	Irritant risk at high dose	[36]
In vivo—UVB-induced skin burn (mouse) ‡	Topical 3% copaiba cream; UVB 0.75 J·cm^−2^	↓ Mechanical allodynia; ↓ thermal hyperalgesia ↓ leukocyte infiltration; no change in dermal thickness; formulation stable ~2 mo	Mixture (copaiba); BCP not isolated	[35]

* Mechanistic anchor (non-BCP) included to support CB_2_–sebocyte plausibility. † Mechanistic (non-BCP) CB_2_ evidence relevant to BCP’s bias. ‡ Mixture study; cannot ascribe effects to BCP alone. Arrows indicate direction of change: upward arrows (↑) denote an increase or upregulation, and downward arrows (↓) denote a decrease or downregulation compared with control. Abbreviations: BCP, β-caryophyllene; CB_2_, cannabinoid receptor type 2; DNCB, dinitrochlorobenzene; EGR1, Early Growth Response 1; FLG, filaggrin; IgE, immunoglobulin E; IL, interleukin; MAPK, mitogen-activated protein kinase; TSLP, thymic stromal lymphopoietin; UVB, ultraviolet B radiation.

**Table 2:** Summary of clinical studies investigating copaiba-derived preparations in dermatology.

Study (Year)	Design/Population	Intervention	Comparator	Duration	Primary Outcomes	Main Outcomes	Limitations/Notes
Waibel et al., 2021 [135]	RCT; adults with abnormal scars	Silicone gel + copaiba oil	Placebo gel	84 days	Manchester Scar Scale (MSS)	Significant improvement in MSS vs. placebo	Multicomponent gel; attribution to BCP not definitive
Cardinelli et al., 2024 [136]	3-arm RCT; hospital patients with skin tears	Polymeric hydrogels (2% or 10% Copaifera multijuga oil)	Vehicle hydrogel	Until closure	Wound healing time, safety	All wounds healed; 2% hydrogel accelerated closure; no adverse events	Multicomponent oleoresin; no BCP-specific PK data
Leite et al., 2023 [137]	Case series; chronic wounds	Topical copaiba oleoresin	None	Variable	Wound healing	Progressive wound closure	Small, uncontrolled; multicomponent extract
da Silva et al., 2019 [138]	RCT; acne vulgaris patients	Copaifera langsdorffii essential oil (topical)	Placebo	Several weeks	Clinical acne severity	Clinical improvement vs. placebo	Multicomponent essential oil; no BCP isolation

Abbreviations: MSS, Manchester Scar Scale; RCT, randomized controlled trial.

**Table 3:** Minimum reporting standards for topical β-Caryophyllene formulations.

Domain	Required Reporting Items	Typical Methods/Notes
Assay/ Degradation	Quantify BCP and BCPO; report peroxide value; perform forced-degradation and photostability studies with preset acceptance limits	GC–MS or GC–FID for BCP/oxide; ICH-style stress protocols
Volatility	Open-system mass-loss testing (~32 °C); headspace GC; include dose-to-skin mass balance	Specify test duration, ambient conditions, and occlusion status
Performance	Finite-dose IVRT/IVPT with defined dose/area, occlusion status, receptor phase (composition), skin source, and temperature	Use human or porcine skin; report membrane integrity checks
Skin Deposition	Depth-resolved tape-stripping or follicular biopsy; specify intact vs. compromised skin	Report number of strips/depth, anatomical site, and recovery efficiency
Packaging and Safety	Oxygen- and light-barrier (airless) packaging; in-use stability under repeated openings; sorption to container parts; TEWL/erythema; HRIPT (where appropriate)	Include container–closure compatibility data; state opening regimen and timepoints

Abbreviations: BCP, β-caryophyllene; BCPO, β-caryophyllene oxide; FID, flame ionization detector; GC, gas chromatography; GC–FID, gas chromatography–flame ionization detection; GC–MS, gas chromatography–mass spectrometry; HRIPT, human repeat insult patch test; ICH, International Council for Harmonization; IVPT, in vitro permeation testing; IVRT, in vitro release testing; TEWL, transepidermal water loss.

**Table 4:** β-Caryophyllene topical delivery systems and enhancer/targeting evidence.

Study (Year)	Study Type/Model	Delivery System/Formulation Type	Core Components/Vehicle	Main Outcomes	Limitations/Notes/Safety
Gushiken et al., 2022 [32]	In vivo rat excision-wound	Conventional emulgel (1% BCP)	Medium-chain triglyceride oil; propylene glycol; polymeric emulsifier	↑ Wound closure; improved inflammatory and antioxidant markers	Simple semisolid system; oxidation monitoring not reported
Alharthi et al., 2023 [142]	Ex vivo permeation; In vivo anti-inflammatory/analgesic	Microemulsion hydrogel (1% BCP)	Isopropyl myristate; propylene glycol; non-ionic surfactants	↑ Dermal permeation (~2×); ↑ retention; ↑ anti-inflammatory and analgesic effects	Direct enhancer evidence; no oxidative stability assessment
Weimer et al., 2022 [103]	Ex vivo permeation; in vivo antiedematogenic	Nanoemulgel	Medium-chain triglyceride oil; non-ionic surfactants; hydrogel base	↑ Dermal permeation; ↑ anti-swelling effect; distinct BCP/BCPO behavior	PK–PD link shown; antioxidant controls not detailed
Ghazwani et al., 2023 [143]	In vitro; ex vivo dermatokinetics	Nanostructured lipid carriers (NLCs)	Solid-lipid matrix with BCP	Controlled release (24 h); ↑ retention (~1.9-fold vs. gel)	In vivo PD and HRIPT not yet performed; oxidation monitoring not reported
Amalraj et al., 2020 [144]	Physicochemical characterization; manufacturability/scale-up	Liposomal BCP (powder, re-dispersible)	Phospholipid vesicles	Improved BCP stability within liposomes; scalable manufacturing validated	Dermal PK/PD not established; efficacy data from oral use only
Tang et al., 2023 [145]	In vitro/ex vivo permeation; in vivo PDT	BCP as penetration enhancer (co-treatment)	Co-administered with 5-aminolevulinic acid in photodynamic therapy	↑ Stratum corneum fluidization; ↑ adjunct active retention; ↑ PDT efficacy	Supports ‘BCP as co-enhancer’ concept; requires finite-dose and oxidation validation

Abbreviations: BCP, β-caryophyllene; HRIPT, human repeat insult patch test; NLCs, nanostructured lipid carriers; PDT, photodynamic therapy; PD, pharmacodynamics; PK, pharmacokinetics; Upward arrows (↑) denote an increase or upregulation compared with control.

**Table 5:** Commercial products containing β-caryophyllene that are marketed for dermatologic applications.

Segment	Brand and Product (Region)	Declared Active(s)/BCP Role	Claims Posture and Regulatory Status *
Acne cleanser (rinse-off)	ISA BEAUTY, Petal’s Flower Acne Facial Wash	No U.S. OTC monograph active declared; BCP present in INCI (fragrance/terpene stack)	“Acne wash” cosmetic positioning; Cosmetic, regulated, not FDA-approved
Acne/rosacea serum	Hemptouch, Skin Perfection Azelaic Serum (EU)	Azelaic acid listed; BCP present as terpene component	Anti-acne/anti-redness; Cosmetic in EU (≤10% azelaic acid); not a U.S. prescription product
Rosacea serum	Rosacea Care, Willowherb Serum with Vitamin K	No monograph drug active; BCP listed as perfuming component	Redness/soothing for rosacea; Cosmetic, regulated, not FDA-approved
Scalp serum (dandruff/itch)	Australian Bodycare, Tea Tree Oil Scalp Serum (EU/UK)	No OTC drug active declared; BCP present with tea tree oil	Dandruff/itchy scalp positioning; Cosmetic, regulated, not FDA-approved
Natural acne/eczema serum	Myrto Natural Cosmetics, Copaiba Blemish Serum (EU)	Copaiba balsam (natural resin rich in BCP)	Acne, psoriasis, skin irritation; marketed as natural cosmetic, not drug
Soothing balm	BareFut, Beta-Caryophyllene Balm (US wellness market)	BCP listed as key active with coconut oil and beeswax	General soothing, relief of irritated/dry skin; Cosmetic, no FDA drug approval
Premium cleansing balm	Eve Lom, Cleansing Balm (EU/US)	Contains clove oil (Eugenia caryophyllus; BCP source) among essential oils	Skin cleansing, texture renewal; Cosmetic, regulated, not FDA-approved
Rosacea/anti-redness care	Rosacea Care, Serum with Willowherb and Vitamin K	BCP present as fragrance/terpene excipient	Anti-redness, soothing; Cosmetic positioning

* Based on publicly available regulatory classifications from FDA, EMA, and regional cosmetic databases at the time of writing; no formal clinical or regulatory endorsement is implied. Inclusion rule: Products with skin-disorder positioning (acne, rosacea, dandruff/itch) where β-caryophyllene/caryophyllene appears on the public INCI/label. Notes: Entries limited to products where β-caryophyllene/caryophyllene is currently listed on public INCI/label pages to minimize error. Products reformulated without BCP, or region-specific variants lacking BCP, were excluded. Abbreviations: BCP, β-caryophyllene; INCI, International Nomenclature of Cosmetic Ingredients; OTC, over the counter.

## Data Availability

Not applicable.

## Abbreviations

The following abbreviations are used in this manuscript:

2-AG	2-arachidonoylglycerol
ABHD6/12	α/β-hydrolase domain–containing proteins 6 and 12
AD	Atopic dermatitis
AEA	N-arachidonoyl ethanolamide (anandamide)
AKT	Protein kinase B
ARE	Antioxidant response element
ARRIVE	Animal Research: Reporting of In Vivo Experiments
BCP	β-caryophyllene
BCPO	β-caryophyllene oxide
CB_1_	Cannabinoid receptor type 1
CB_2_	Cannabinoid receptor type 2
CBD	Cannabidiol
CREB	cAMP response element–binding protein
CNS	Central nervous system
COX-2	Cyclooxygenase-2
DAGLα/β	Diacylglycerol lipase α/β
eCBs	Endocannabinoids
ECS	Endocannabinoid system
EDC	Epidermal differentiation complex
EGR1	Early growth response protein 1
EMA	European Medicines Agency
ERK	Extracellular signal–regulated kinase
FAAH	Fatty acid amide hydrolase
FABP5/7	Fatty acid binding proteins 5/7
FDA	U.S. Food and Drug Administration
FEMA	Flavor and Extract Manufacturers Association
FLG	Filaggrin
GC–FID	Gas Chromatography–Flame Ionization Detection
GC–MS	Gas Chromatography–Mass Spectrometry
GMP	Good Manufacturing Practice
GPCRs	G-protein-coupled receptors
GPR55	G-protein-coupled receptor 55
GRAS	Generally Recognized as Safe
GSH	Glutathione
HO-1	Heme oxygenase-1
HRIPT	Human repeat insult patch test
IL	Interleukin
INCI	International Nomenclature of Cosmetic Ingredients
IVL	Involucrin
IVPT	In vitro permeation testing
IVRT	In vitro release testing
JNK	c-Jun N-terminal kinase
LOR	Loricrin
LPS	Lipopolysaccharide
MAGL	Monoacylglycerol lipase
MAPKs	Mitogen-activated protein kinases
MDA	Malondialdehyde.
MHC	Major histocompatibility complex
MMPs	Matrix metalloproteinases
NAPE-PLD	N-acyl-phosphatidylethanolamine phospholipase D
NF-κB	Nuclear factor kappa-light-chain-enhancer of activated B cells
NLCs	Nanostructured lipid carriers
Nrf2	Nuclear factor erythroid 2–related factor 2
PD	Pharmacodynamics
PI3K	Phosphoinositide 3-kinase
PK	Pharmacokinetics
PPARγ	Peroxisome proliferator-activated receptor gamma
PRISMA	Preferred Reporting Items for Systematic Reviews and Meta-Analyses
RhoA	Ras homolog family member A
RIFM	Research Institute for Fragrance Materials
RoB 2	Risk of Bias tool (for clinical trials)
ROS	Reactive oxygen species
SOD	Superoxide dismutase
STAT	Signal transducer and activator of transcription
TEER	Transepithelial electrical resistance
TEWL	Transepidermal water loss
Th2	T helper 2
TIMPs	Tissue inhibitors of metalloproteinases
TNF-α	Tumor necrosis factor-alpha
TRP	Transient receptor potential channels
TRPA1	Transient receptor potential ankyrin 1
TRPC6	Transient receptor potential canonical 6
TRPM8	Transient receptor potential melastatin 8
TRPV1	Transient receptor potential vanilloid 1
TRPV3	Transient receptor potential vanilloid 3
TRPV4	Transient receptor potential vanilloid 4
TSLP	Thymic stromal lymphopoietin
UVB	Ultraviolet B radiation
Wnt	Wingless-related integration site pathway
